# Evaluating the Fat Distribution in Idiopathic Intracranial Hypertension Using Dual-Energy X-ray Absorptiometry Scanning

**DOI:** 10.1080/01658107.2017.1334218

**Published:** 2017-06-20

**Authors:** Catherine Hornby, Hannah Botfield, Michael W. O’Reilly, Connar Westgate, James Mitchell, Susan P. Mollan, Konstantinos Manolopoulos, Jeremy Tomlinson, Alexandra Sinclair

**Affiliations:** a Institute of Metabolism and Systems Research, College of Medical and Dental Sciences, University of Birmingham, Birmingham, United Kingdom; b Centre for Endocrinology, Diabetes and Metabolism, Birmingham Health Partners, Birmingham, United Kingdom; c Department of Neurology, University Hospitals Birmingham NHS Foundation Trust, Birmingham, United Kingdom; d Birmingham Neuro-Ophthalmology Unit, Ophthalmology Department, University Hospitals Birmingham NHS Trust, Queen Elizabeth Hospital Birmingham, Birmingham, United Kingdom; e Oxford Centre for Diabetes, Endocrinology and Metabolism (OCDEM), University of Oxford, Oxford, United Kingdom

**Keywords:** DEXA, idiopathic intracranial hypertension, obesity

## Abstract

Idiopathic intracranial hypertension (IIH) is strongly associated with obesity. We aimed to utilise dual-energy X-ray absorptiometry (DEXA) to characterise fat distribution, and to evaluate change in fat mass and distribution following weight loss. IIH patients (*n* = 24) had a similar fat distribution to body mass index (BMI)– and gender-matched obese controls (*n* = 47). In the IIH cohort, truncal fat mass correlated with lumbar puncture pressure. Weight loss in IIH patients resulted in a significant reduction in disease activity and fat mass, predominantly from the truncal region (−4.40 ± 1.6%; *p* = 0.008) compared with the limbs (+0.79 ± 6.5%; *p* = 0.71). These results indicate that, contrary to previous studies using waist-hip ratios, IIH adiposity is centripetal, similar to simple obesity. Future studies should establish the risk of the metabolic syndrome and the role of adipose tissue depot–specific function in IIH.

## Introduction

Idiopathic intracranial hypertension (IIH) is a condition of raised intracranial pressure (ICP) of unknown cause.^1^ The presentation is variable but typically includes chronic headaches and, in the majority, papilloedema that can result in visual decline.^2^ IIH has a striking phenotype of young, obese women of childbearing age.^2,3^ There is clearly a strong association between the condition and obesity, which is seen in over 90% of IIH patients,^3^ and weight loss has been shown to resolve symptoms and improve clinical parameters of IIH. However, not all obese patients develop IIH, and it is not known whether the pattern of body fat distribution could contribute to development of disease.^4^ This is of particular interest, given the well-known association between abdominal (upper) and gluteofemoral (lower) body fat ratios and the risk of cardiometabolic diseases.^5,6^

We aimed to characterise the fat distribution of women with IIH compared with controls using the gold-standard dual-energy X-ray absorptiometry (DEXA) scanning technique, which has not been previously used to evaluate fat mass, fat distribution, or percentage body fat in IIH. This technique measures fat mass and distribution for total body and regional areas and is significantly more accurate than waist-hip ratios, which are prone to inaccuracy due to inconsistent identification of the waist in obese patients.^7^

## Materials and methods

Patients with active IIH were recruited as previously described, and the control cohort was composed of patients with simple obesity.^8,9^

DEXA was performed using a total-body scanner (QDR 4500; Hologic, Bedford, MA, USA), as previously described.^9,10^ The scans were administered by a clinical scientist and trained radiographer. Patients with metal prosthetics or implants were included, and tissue overlying the prosthesis was excluded from analysis. Scans were checked for accuracy of fields of measurement. Regional fat mass was analysed as described previously.^9–11^ The precision of total fat mass measures in terms of coefficients of variation (CV) was less than 3%, and for regional fat analyses it was less than 5%. Regional fat data were expressed as a percentage of the total body fat. Both the IIH and control cohorts were analysed on the same DEXA scanner over the same time period.

Lipid profiling (cholesterol and triglycerides) were performed in both IIH and control cohorts to assess risk factors for the metabolic syndrome. Total cholesterol and triglycerides were measured following an overnight fast from midnight.

Furthermore, the IIH cohort also underwent a weight loss intervention, during which they lost on average 15% of body weight, as previously described.^8^ The intervention used a previously validated, low-energy total meal replacement liquid diet (Lipotrim; Howard Foundation, Cambridge, UK), providing 425 kcal/day, for 3 months.^12^ Patients were unable to consume additional food but were instructed to drink at least 2 L of fluid a day. This was followed by further DEXA scanning. We therefore also assessed the influence of therapeutic weight loss on fat distribution in IIH. A small subset of the control cohort were evaluated using the same pre– and post–weight loss protocol.^13^ This cohort underwent the same diet intervention as the IIH patients.

Measures of IIH severity were measured before and after weight loss intervention. Intracranial pressure was measured using lumbar puncture (LP), with measurements taken with the patient lying in the left lateral position, with legs extended greater than 90° at the hip. Adequate time was taken to ensure a stable pressure reading. Papilloedema was assessed using optical coherence tomography (Stratus OCT V4.0.1; Carl Zeiss, Meditec, Welwyn Garden City, UK), peripapillary circle scan of the average retinal nerve fibre layer thickness (µm), and masked papilloedema grading of fundus photographs by a neuro-ophthalmologist (Frisen grading^14^).

This study was approved by the Dudley local research ethics committee (06/Q2702/64) and the South Birmingham Local Research Ethics Committee (04/Q2707/278).

### Statistical analysis

Statistical analysis was performed using SPSS versions 14 and 15 (SPSS Inc., Chicago, IL, USA) and GraphPad Prism 7 for Mac OS X (GraphPad Software Inc., La Jolla, CA, USA). Data are reported using mean and standard deviation. Spearman’s rank correlation coefficient was used for assessing correlation of non-parametric data. Correlation data were subject to multiple comparisons and consequently a Bonferroni correction was applied. Mann-Whitney *U* tests were used for assessment of non-parametric data. Data from the right and left eyes yielded analogous results and correlated significantly; consequently, data from the right eye only are listed. The level at which results were judged significant was *p* < 0.05.

## Results

IIH patients (*n* = 24) were compared with a gender- and body mass index (BMI)–matched control cohort (*n* = 47) (mean BMI was 36.2 ± 3.7 kg/m^2^ in IIH and 34.5 ± 4.3 kg/m^2^ in controls; *p* = 0.08). Cohorts were not matched for age, with the control cohort being significantly older than the IIH cohort (49.1 ± 6.9 vs. 32.8 ± 8.6 years, respectively). Waist measurements showed no significant difference between the two groups (106.5 ± 9.7 cm in the IIH group and 104.2 ± 9.7 cm in controls; *p* = 0.343).

**Figure 1.**
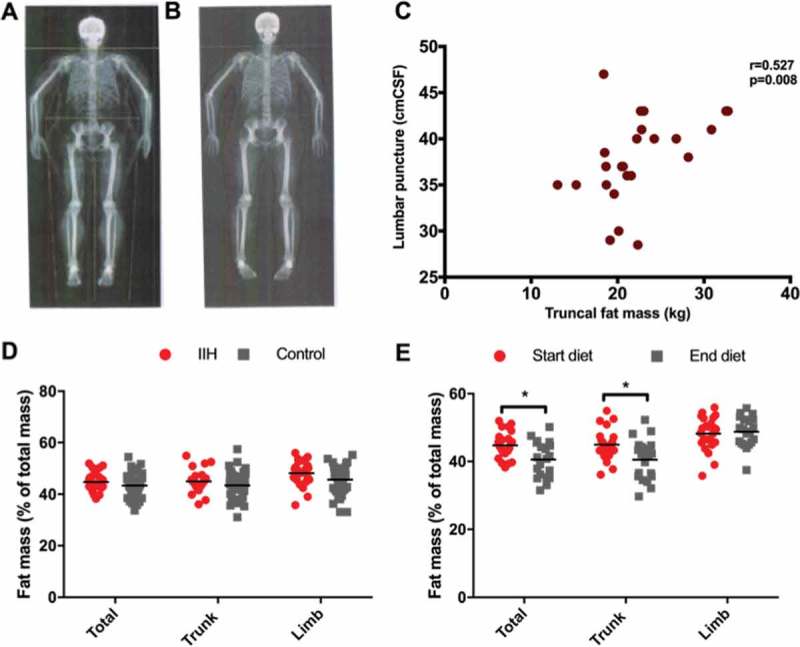
Fat distribution in IIH and response to weight loss. Example DEXA imaging before (A) and after (B) weight loss. (C) Correlation between truncal fat mass and lumbar puncture (LP) pressure. (D) Fat mass distribution in IIH compared with controls. (E) Percentages of fat mass in IIH before and after weight loss (*p < 0.05).

The DEXA scanner was able to accommodate all patients enrolled in the study. The DEXA scanning illustrated no difference in trunk and limb fat distribution in IIH patients compared with women with simple obesity (Figure 1). Trunk fat in IIH was 20.5 ± 3.2 kg compared with 19.9 ± 5.2 kg in controls (*p* = 0.19), whereas limb fat in IIH was 20.5 ± 5.0 kg and in controls was 18.3 ± 4.3 kg (*p* = 0.14). Although the ages were not matched, age did not appear to affect results, as there was no difference in fat distribution between IIH and control subjects following multivariate regression analysis, taking into account age as a covariate. Interestingly, truncal fat mass correlated with LP pressure in the IIH cohort (*r* = 0.527, *p* = 0.008; Figure 1). There was no significant correlation between LP pressure and BMI or limb fat mass; total fat mass and waist circumference showed a trend towards an association, but this failed to reach statistical significance set to *p* = 0.01 once a Bonferroni correction was applied (Table 1). There was no correlation between papilloedema (measured by average retinal nerve fibre layer thickness on optical coherence tomography and Frisen grading) and the anthropological measures (BMI and waist circumference) and DEXA distribution data (total, truncal, and limb fat mass).

**Table 1.** Correlation analysis of anthropological measures and DEXA scanning fat distribution data against lumbar puncture (LP) pressure and papilloedema as measured by optical coherence tomography (OCT) peripapillary circle scan of the average retinal nerve fibre layer thickness (µm) in the right eye.

IIH measure	BMI (kg/m^−2^)	Waist circumference (cm)	Total fat mass (g)	Trunk fat (g)	Limb fat (g)
LP pressure (cm CSF)	*r* = 0.018	*r* = 0.461	*r* = 0.510	*r* = 0.527	*r* = 0.397
	*p* = 0.934	*p* = 0.023	*p* = 0.011	*p* = 0.008**	*p* = 0.055
OCT (µm)	*r* = 0.199	*r* = 0.215	*r* = 0.276	*r* = 0.183	*r* = 0.314
	*p* = 0.351	*p* = 0.312	*p* = 0.192	*p* = 0.391	*p* = 0.1335

*Note*. IIH = idiopathic intracranial hypertension. The level of statistical significance was adjusted to *p* < 0.01 following a Bonferroni correction to account for multiple comparisons. The only significant correlation was between LP pressure and truncal fat.

We previously demonstrated significant weight loss that corresponded to improvement in LP pressure and papilloedema.^8^ The DEXA scanning revealed that although fat mass reduced significantly from all areas following the diet, when analysing change in fat mass as a percentage of total body fat mass, the fat mass was predominantly lost from the truncal region (−4.40 ± 1.6%; *p* = 0.008), compared with limbs (+0.79 ± 6.5%; *p* = 0.71; see Figure 1). However, these data demonstrate that the change in fat mass and distribution measured by DEXA did not correlate with changes in ICP or papilloedema.

A small subset of the control cohort also underwent the weight loss intervention and post–weight loss DEXA scanning (*n* = 13). Controls lost a similar amount of weight compared with IIH patients (weight loss in the IIH cohort undergoing DEXA scanning was −15.2 ± 3.0 kg compared with 13.3 ± 4.4 kg in controls; *p* = 0.775; see Table 2). Fat mass was lost in a similar distribution to that seen in IIH (truncal fat mass lost in IIH was −4.40 ± 21.6 kg compared with −4.9 ± 2.4 kg in controls; *p* = 0.870; Table 2).

**Table 2.** Demographics of the subset of IIH and obese control patients undergoing the weight loss intervention.

Characteristic	IIH	Control	*p*-value
Number	22	13	
Gender (% female)	100	100	
Age (years)	34.4 ± 9.2	40.4 ± 13.5	0.224
Weight (kg)	101.3 ±16.5	100.4 ± 12.5	0.863
BMI (kg/m^2^)	37.9 ± 4.9	36.5 ± 4.8	0.369
Waist (cm)	111.1 ± 10.4	104.3 ± 7.8	0.043
Change following weight loss			
Weight (kg)	−15.2 ± 3.0	−13.3 ± 4.4	0.775
BMI (kg/m^2^)	−5.8 ± 3.0	−4.8 ± 1.5	0.555
Waist (cm)	−9.4 ± 5.9	−10.4 ± 5.5	0.649
DEXA			
Total fat mass (kg)	−9.10 ± 4.7	−9.3 ± 3.4	0.957
Total fat (%)	−4.1 ± 2.7	−4.0 ± 2.4	0.986
Trunk fat mass (kg)	−4.40 ± 21.6	−4.9 ± 2.4	0.870

*Note*. BMI = body mass index; DEXA = dual-energy X-ray absorptiometry; IIH = idiopathic intracranial hypertension. Values are shown as mean ± standard deviation.

## Discussion

In summary, we have used DEXA scanning to objectively quantify fat distribution in IIH and find that it is centripetal, mirroring that of simple obesity. Previous studies, from a single study centre, using waist-hip ratios, have identified preferential lower body fat accumulation in IIH.^15,16^ This has not been our experience. These previous findings may be in relation to the tendency for gluteofemoral fat to “accumulate” in the typical female fat distribution, as seen in previous studies.^5^ Clinical resolution of IIH occurs following a loss of truncal fat, potentially implicating central obesity in the pathogenesis of IIH. We found that this degree and pattern of weight loss was similar to that in obese controls, although as the number of controls is small, this analysis is limited. The importance of truncal fat is in line with many previous studies showing a strong correlation between abdominal fat accumulation and adverse health outcomes, such as an increased cardiovascular risk, dyslipidaemia, insulin resistance, and type 2 diabetes.^17–19^ Importantly, total abdominal fat mass, as measured by waist circumference or DEXA, is determined by the mass of two distinct adipose tissue depots, the subcutaneous and the visceral depot, with the latter particularly contributing to the adverse effects of central obesity.^20,21^ Weight loss is associated with overall reductions in total body fat mass, whereby there appears to be gender- and depot-specific differences.^22,23^ Our data are in line with previous studies in women, showing reductions in abdominal fat mass following dietary intervention.^24,25^

Of interest, we found that truncal fat mass, but not BMI, was significantly correlated with LP pressure. In studies of patients with mixed neurological conditions, obesity as measured by BMI did not correlate with LP pressure, but truncal adiposity has not been widely evaluated.^26^ We did not find an association between change in DEXA fat distribution (total, truncal, or limb fat), BMI, or waist circumference and changes in LP pressure or papilloedema.

## Conclusion

The exact pathophysiological mechanism underpinning the relationship between body fat distribution, the female gender predominance, and the development of IIH remains unknown. This study highlights that patients with IIH have a similar distribution of fat mass to patients with simple obesity. Truncal fat may have pathological importance in IIH, as we noted that truncal fat mass was correlated with LP pressure. Additionally, resolution of IIH following weight loss was associated with preferential loss of adiposity from the truncal region. Future research into the role of adipose tissue depot–specific function, including the secretion of adipokines and inflammatory factors, in regulating ICP is required. Additionally, future evaluation to establish if IIH patients have an increased risk of metabolic syndrome will have implications for long-term morbidity.
